# Curcumin activates the p38MPAK-HSP25 pathway *in vitro *but fails to attenuate diabetic nephropathy in DBA2J mice despite urinary clearance documented by HPLC

**DOI:** 10.1186/1472-6882-10-67

**Published:** 2010-11-12

**Authors:** Jun Ma, Lynetta Phillips, Ying Wang, Tiane Dai, Janine LaPage, Rama Natarajan, Sharon G Adler

**Affiliations:** 1 Los Angeles Biomedical Research Institute at Harbor-UCLA Medical Center, Division of Nephrology and Hypertension, 1124 W Carson St, Torrance, CA 90502, USA

## Abstract

**Background:**

Curcumin has anti-inflammatory, anti-oxidant, and anti-proliferative properties, and depending upon the experimental circumstances, may be pro- or anti-apoptotic. Many of these biological actions could ameliorate diabetic nephropathy.

**Methods/Design:**

Mouse podocytes, cultured in basal or high glucose conditions, underwent acute exposure to curcumin. Western blots for p38-MAPK, COX-2 and cleaved caspase-3; isoelectric focusing for HSP25 phosphorylation; and DNase I assays for F- to G- actin cleavage were performed for *in vitro *analyses. *In vivo *studies examined the effects of dietary curcumin on the development of diabetic nephropathy in streptozotocin (Stz)-induced diabetes in DBA2J mice. Urinary albumin to creatinine ratios were obtained, high performance liquid chromatography was performed for urinary curcuminoid measurements, and Western blots for p38-MAPK and total HSP25 were performed.

**Results:**

Curcumin enhanced the phosphorylation of both p38MAPK and downstream HSP25; inhibited COX-2; induced a trend towards attenuation of F- to G-actin cleavage; and dramatically inhibited the activation of caspase-3 in *vitro*. In curcumin-treated DBA2J mice with Stz-diabetes, HPLC measurements confirmed the presence of urinary curcuminoid. Nevertheless, dietary provision of curcumin either before or after the induction of diabetes failed to attenuate albuminuria.

**Conclusions:**

Apart from species, strain, early differences in glycemic control, and/or dosing effects, the failure to modulate albuminuria may have been due to a decrement in renal HSP25 or stimulation of the 12/15 lipoxygenase pathway in DBA2J mice fed curcumin. In addition, these studies suggest that timed urine collections may be useful for monitoring curcumin dosing and renal pharmacodynamic effects.

## Background

Diabetic nephropathy (DN) remains the commonest cause of end stage renal disease. Albuminuria, the cardinal clinical feature of DN, is induced by mechanisms undergoing reappraisal [1], but which primarily involve podocyte pathology, along with alterations in the glomerular basement membrane (GBM), endothelium, mesangium, and renal tubule cells [2-8]. Podocyte effacement is closely aligned with albuminuria and reflects, at least in part, actin cytoskeletal rearrangement.

Heat shock proteins (HSP) are ubiquitously expressed across virtually all phyla. Classified by molecular weight, HSPs influence key biological processes such as cell division and cell survival [9], differentiation, actin cytoskeleton regulation, and resistance to injury from reactive oxygen species (ROS), and other cell stressors [10]. HSP25, the rodent homolog of human HSP27, is phosphorylated by upstream p38 mitogen-activated protein kinase (p38MAPK). Phosphorylated HSP25 plays a key role in the regulation of actin cytoskeletal dynamics [11-17]. We previously showed *in vitro *that short-term incubation of podocytes in medium with a high glucose concentration (up to 4 hours) resulted in phosphorylation of p38MAPK and downstream HSP25, associated with maintenance of the actin cytoskeleton. Incubation of podocytes in high glucose medium for as briefly as 4 hours with a p38MAPK inhibitor attenuated downstream HSP25 phosphorylation, inducing F- to G-actin cleavage, and cytoskeletal disruption. We previously showed *in vitro *that short-term incubation of podocytes in medium with a high glucose concentration resulted in phosphorylation of p38MAPK and downstream HSP25, associated with maintenance of the actin cytoskeleton. Incubation of podocytes in high glucose medium for hours, or incubation with a p38MAPK inhibitor, attenuated downstream HSP25 phosphorylation, inducing F- to G-actin cleavage, and cytoskeletal disruption. *In vivo*, we showed that acutely after the induction of diabetes with streptozotocin (Stz-DM) in rats, there is coordinated activation of the glomerular p38MAPK-HSP25 pathway, in association with maintenance of the podocyte actin cytoskeleton and normoalbuminuria. However, with chronicity of Stz-DM, pathway activation declines, F-actin cleavage generates G-actin monomers, and podocyte effacement and albuminuria occur [18]. These associations generated the hypothesis that early activation of the p38MAPK-HSP25 pathway might be a functional adaptation that maintained podocyte structure and function and prevented albuminuria in response to the glucose stressor. Based on these observations, we posited that therapies that prolonged the activation of the p38MAPK-HSP25 pathway would attenuate albuminuria.

Curcumin (diferuloylmethane) is one of the most commonly used spices in the world. In numerous cell types, exposure to curcumin has been shown to increase HSPs *in vitro *[14-17,19-22]. We performed experiments to determine whether curcumin activates the p38MAPK -HSP25-actin cytoskeletal pathway in glucose-stimulated podocytes *in vitro*, and whether it attenuates diabetic nephropathy (DN) *in vivo *in mice in whom feeding was begun either before or 1 week after the induction of Stz-DM.

## Methods & Design

### Podocyte Culture

Conditionally immortalized mouse podocytes (Pods), carrying a thermosensitive SV40 transgene, were obtained from Dr. Peter Mundel and cultured as described with minor modifications [23]. Briefly, PODs proliferated at 33°C (permissive conditions) in RPMI 1640 media supplemented with 5.5 mM glucose, 10% fetal bovine serum (FBS), γ-IFN (tapered from 50 U/ml to 10 U/ml), and 1% penicillin/streptomycin/amphotericin B (Invitrogen, Carlsbad, CA). Pods were grown in collagen-coated flasks in a humidified atmosphere of 95% air and 5% CO_2_. Cells were then thermoshifted to 37° and allowed to differentiate for 14 days without γ-IFN (nonpermissive conditions) with media changed on alternate days. Pods between 4-8 passages were used for all experiments.

Cells were serum starved in RPMI with 0.4% FBS for 24 h. Dose and time-course experiments were performed to determine optimal conditions for the experiments. Curcumin (Cur, generously donated by Sabinsa Corporation, Piscataway, NJ) was dissolved in 100% ethanol at a stock concentration of 10 mM and further diluted to experimental concentrations ranging from 1 μM to 100 μM in RPMI. In dose-response preliminary *in vitro *studies, 30 μM Cur demonstrated the most robust HSP25 signaling activation and was used for all experiments. Cur at 100 μM induced cell death (data not shown). The effects of Cur on the phosphorylated p38 mitogen-activated protein kinase (pp38MAPK) - phosphorylated HSP25/27 (pHSP25/27) signaling pathway in the presence and absence of glycemic stress were assessed with the following treatment groups: 1) 5.5 mM glucose for 60-70 min (normal glucose, NG); 2) 5.5 mM glucose with 30 uM Cur for 60-70 min (NG+Cur); 3) 5.5 mM glucose for 60 min immediately replaced by 30 mM glucose for 10 min (high glucose, HG); 4) 5.5 mM glucose with 30 μM Cur for 60 min immediately replaced by 30 mM glucose with 30 μM Cur for 10 min (HG+Cur); and 5) 5.5 mM glucose + mannitol to achieve iso-osmolarity (5.5 + 24.5 mM) (NG + M). The HG/Cur treatment used in isoelectric focusing was performed under the following conditions: 5.5 mM glucose with 30 μM Cur for 60 min immediately replaced by 30 mM glucose for 10 min. Published work from our lab [18] showed that HG for 10 min induced significant increases in pp38MAPK and pHSP25 in Pods. Thus, a 10 min HG treatment period was used in the current study. Cells were harvested in RIPA or urea buffer (see below) following treatments.

### Western Blot Analysis

Following experimental treatments, cells were washed with ice cold phosphate-buffered saline (PBS) and harvested in RIPA buffer (1 × PBS, 1% nonidet P-40, 0.5% sodium deoxycholate, 0.1% sodium dodecyl sulfate, 20 mM sodium fluoride) with proteinase and phosphatase inhibitor cocktails 1 and 2 (Sigma-Aldrich, St Louis, MO, USA). Cells were sonicated, centrifuged at 10,000 × *g *for 10 min at 4°C, and cell lysates stored at -20°C until use. Protein concentration in cell lysate was measured using Protein Assay Dye Reagent (Bio-Rad Laboratories, Hercules, CA) and known bovine serum albumin (BSA) concentrations as standards. Supernatants containing 50-100 μg protein were loaded onto 7-15% gradient sodium dodecyl sulfate (SDS)-polyacrylamide gels. Following electrophoresis, proteins were transferred overnight onto nitrocellulose membranes (GE Osmonics Labstore, Minnetonka, MN) and blocked with 5% milk or 5% BSA in tris-buffered saline solution with 0.2% Tween 20.

Membranes were probed with the following antibodies: HSP25 (Stressgen, Victoria, BC, Canada), total p38MAPK, phospho-p38MAPK and cleaved caspase-3 (Cell Signaling Technology, Inc. Danvers, MA, USA), cyclooxygenase-2 (COX-2)(Santa Cruz Biotechnology, Santa Cruz, CA, USA), glyceradehyde-3-phosphate dehydrogenase (GAPDH, Fitzgerald Industries International, Inc., Concord, MA, USA), goat anti-mouse IgG (Santa Cruz Biotechnology), goat anti-rabbit IgG (Cell Signaling Technology, Inc.) and mouse anti-goat IgG (Santa Cruz Biotechnology). Western blots were incubated in commercial enhanced chemiluminescence reagents (Pierce Biotechnology, Inc., Rockford, IL) and exposed to photographic film. Densitometry was quantified using AlphaDigiDoc 1000 software (Alpha Innotech Corporation, San Leandro, CA).

### Isoelectric Focusing for HSP25

Isoelectric focusing (IEF) was performed to measure concentrations of phosphorylated HSP25 as described previously [2,3]. All samples for IEF were solubilized in urea buffer (9 M urea, 2% nonidet P-40, 0.005% β-mercaptoethanol) at the time of cell harvesting and stored at -20°C until use.

### DNase 1 inhibition assay for the measurement of F/G actin ratio

Pod F- and G-actin were measured using the methods of others [24,25] and as we previously utilized [18]. Once solubilized in lysis buffer, lysate was added to DNAse I solution (0.1 mg/mL bovine pancreas DNase I in 50 mM Tris/HCl, 10 mM phenylmethylsulfonyl fluoride, 0.5 mM CaCl_2_, pH 7.5) and DNA solution (40 μg/mL calf thymus DNA type 1 in 100 mM Tris/HCl, 4 mM MgSO_4_, 1.8 mM CaCl_2_, pH 7.5). DNase I activity was monitored at 260 nm. Actin was measured using a standard curve for inhibition of DNase I activity using rabbit skeletal muscle G-actin (Sigma-Aldrich). Linearity was established between 25 and 70% inhibition of DNase I activity. For total actin, lysates were diluted with lysis buffer and incubated on ice with an equal volume of guanidine/HCl buffer to depolymerize F-actin to monomeric G-actin. F-actin was calculated as the difference between total and G-actin.

### Experimental Animals

Diabetes mellitus (DM) was induced in male 20-22 gm two-month old DBA/2J mice (Jackson Laboratories, Bar Harbor, Maine, USA) by injecting a daily dose of streptozotocin (Stz, 50 mg/kg, i.p., prepared in 0.05 M sodium citrate buffer) for 5 consecutive days. Age-matched control mice received only sodium citrate buffer. Diabetes was confirmed by fasting blood glucose levels one week after the 5^th ^daily Stz injection. Control and DM mice were placed on custom-prepared diets (Purina Mills LLC TestDiet^® ^Division, Richmond, IN) that differed only in Cur content. The diet compositor, and initial dosing assignments, were chosen based on the prior experience of the Purina Mills LLC Test Diet Division, who prepared identical diets for a therapeutically successful study using Cur for a mouse model of Alzheimer disease [26]. Two studies were performed. In Experiment 1, Cur feeding at 5,000 ppm (Cur_5,000 ppm_) began one week after the last Stz injection, at the time the diagnosis of DM was confirmed. Due to the inability to show benefit from Cur in Experiment 1, in Experiment 2, pre-feeding of Cur_5,000 ppm _or Cur_7,500 ppm _was begun prior to DM induction by Stz injections.

In Experiment 1, non-diabetic (noDM) or DM mice were assigned to one of the following diets at the time the DM was confirmed in the Stz-injected group (Day 0): 1) control chow with 0 ppm Cur (n = 8 for noDMCur_0_; n = 11 for DMCur_0_); 2) test chow with Cur_5,000 ppm _(n = 10 for noDMCur_5000_; n = 6 for DMCur_5000_). For each mouse, food and water intake were measured. On days 9 and 15 of the study, mice were placed in individual metabolic cages with 5% dextrose in water for an overnight collection to measure urinary albumin (ELISA Albuwell M Kit Stock no. 1011, Exocell Inc., Philadelphia, PA), creatinine (Creatinine Companion Stock no. 1012, Exocell Inc., Philadelphia, PA), the arachidonic acid metabolite 12-hydroxytetraenoic acid (12-HETE), and Cur and its metabolites by high performance liquid chromatography (HPLC). For urine albumin/creatinine ratio, data was expressed as log_10 _in order to achieve a normal distribution.

In Experiment 2, mice were randomly assigned to receive a control or Cur diet one week prior to Stz injections. Mice were then injected with Stz daily for 5 days as described above. DM was ascertained one week after the last Stz injection (Day 0), and then again in steady state from weeks 5-7, and in some mice specially maintained for glycemic monitoring, up to 11 weeks. The following experimental conditions were compared: 1) control chow with 0 ppm Cur (n = 5 for noDMCur_0; _n = 5 for DMCur_0_); 2) test chow with Cur_5,000 ppm _(n = 6 for noDMCur_5000; _n = 7 for DMCur_5000_); or 3) test chow Cur_7,500 ppm _(n = 6 for noDM Cur_7500; _n = 5 for DMCur_7500_). Timed urine collections were made on weeks 2, 4, and 7 for urine albumin and creatinine measurements. All studies were performed under a protocol approved by the Los Angeles Biomedical Research Institute Animal Use Committee. Mice were sacrificed by exsanguination under general anesthesia.

### Measurement of Urinary 12-HETE and Cur

Urinary 12-HETE was measured by enzyme immunoassay (12-HETE EIA, 12(S)-HETE Correlate EIA Kit Catalog no. 900-050, Assay Designs Inc., Ann Arbor, MI). Cur and its metabolites (total curcuminoid) were measured at the Nutrition Core Research Laboratory at the University of California at San Diego using HPLC at a wavelength of 262 nm by methods similar to those previously reported [27]. However, an interfering background peak that co-eluted with urine total curcuminoid at 262 nm was identified. The mean OD values measured at 262 nm from the urine of mice receiving control diet Cur_0 _were subtracted from the urine results from mice receiving Cur in the diet in order to compensate for the presence of the interfering substance.

### Statistical Analysis

Analysis of variance followed by Student's *t *test was utilized for analysis of all results. Statistical calculations were performed using StatMost (Salt Lake City, UT). Data are expressed as mean ± SEM. Significance is assigned at the p < 0.05 level.

## Results and discussion

### Cur stimulates phosphorylation of p38MAPK and HSP25 in cultured podocytes

The ability of Cur to stimulate phosphorylation of p38MAPK and HSP25 in both normal glucose (NG, 5.5 mM) and high glucose (HG, 30 mM) media was assessed. To determine optimal experimental conditions, a pilot dose-dependent titration experiment was performed exposing Pods to Cur concentrations ranging from 1-100 μM. 30 μM Cur (Cur _30 μM_) stimulated total HSP25 protein expression the most (data not shown) and was therefore used for all subsequent experiments. A time-dependent titration further demonstrated that treating cells with Cur for 60-70 min stimulated HSP25 protein expression (data not shown). Our published work showed that incubating Pods in HG for up to 24 hrs stimulated the phosphorylation of p38MAPK and HSP25 while maintaining the actin cytoskeleton [18]. We performed all subsequent experiments under the conditions selected from these initial studies. Mannitol served as an iso-osmotic control.

**Figure 1:**
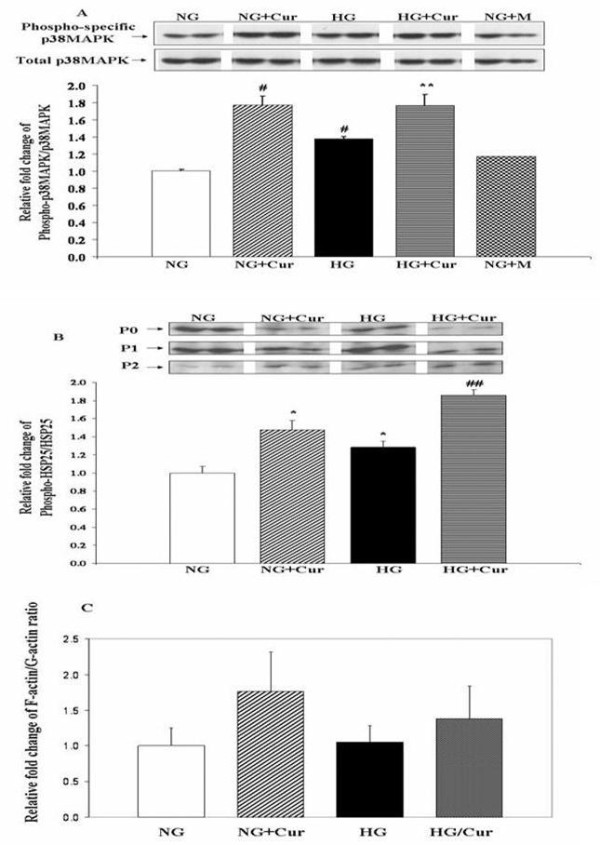
**Curcumin activates p38MAPK and phosphorylates HSP25 in cultured Pods**. 5.5 mM glucose (NG), 5.5 mM glucose+30 μM curcumin (NG+Cur), 30 mM glucose (HG), 30 mM glucose+30 μM curcumin (HG+Cur), 5.5 mM glucose+24.5 mM mannitol (NG+M). (a) Representative Western blots of phospho-specific p38MAPK (pp38MAPK) and quantitative evaluation of pp38MAPK relative to total p38MAPK (p38MAPK) by densitometric analysis. (b) Representative IEF separating total HSP25 into its nonphosphorylated isoform (P0), mono- (P1), and bi-phoshorylated (P2) isoforms, Western blots of HSP25, and quantitative evaluation of relative phosphorylated HSP25 isoforms to total HSP25 ((P1 + P2)/(P0 + P1 + P2)) by densitometry analysis. Mannitol values (n = 2) are not displayed but were similar to NG. (c) DNAse I assay of F-actin/G-actin ratios. All data expressed as mean ± SEM (n = 3). *P < 0.05 compared with NG; #P < 0.01 compared with NG; **P < 0.05 compared with HG; ##P < 0.01 compared with HG.

Cur significantly increased podocyte pp38MAPK 1.8-fold when added to NG media (NG+ Cur_30 μM_, 1.77 ± 0.10 vs. NG, 1.00 ± 0.02; P < 0.01; Figure 1a). As anticipated, podocyte pp38MAPK was significantly higher in HG compared to NG, but Cur_30 μM _further increased pp38MAPK even when added to HG (1.76 ± 0.13 versus 1.38 ± 0.03; P < 0.05). Thus, when added to either NG or HG, Cur_30 μM _exposure further increased p38MAPK activation by a similar degree. Mannitol did not replicate the p38MAPK activation induced by HG, indicating an effect occurring independent of osmolarity.

Activation of p38MAPK phosphorylates downstream HSP25/27. Thus, podocyte exposure to Cur_30 μM _induced HSP25 biphosphorylation as demonstrated by isoelectric focusing (IEF) (Figure 1b). Cur_30 μM_, when added to NG, significantly increased the biphosphorylated HSP25/total HSP25 ratio by ~1.5-fold (1.00 ± 0.07 (NG) vs. 1.47 ± 0.11 (NG+ Cur_30 μM_), P < 0.01; Figure 1b). As anticipated, the biphosphorylated HSP25/total HSP25 ratio also significantly increased when Cur_30 μM _was added to HG medium (1.28 ± 0.07 (HG) vs 1.86 ± 0.061(HG+ Cur_30 μM_), P < 0.05). Mannitol did not affect pHSP25 phosphorylation (data not shown).

Curcumin's effect on the preservation of Pod actin cytoskeleton was directly examined (Figure 1c). There was a trend towards increased filamentous to monomeric globular actin ratio (F/G-actin) in Pods receiving Cur (1.8-fold in NG, 1.3-fold in HG). These increases in F/G-actin fell short of statistical significance. Collectively, these data demonstrate that Cur activates the Pod p38MAPK-HSP25 signaling pathway by phosphorylation *in vitro *under both NG and HG conditions.

### Cur prevents caspase-3 activation and inhibits COX-2 expression

**Figure 2:**
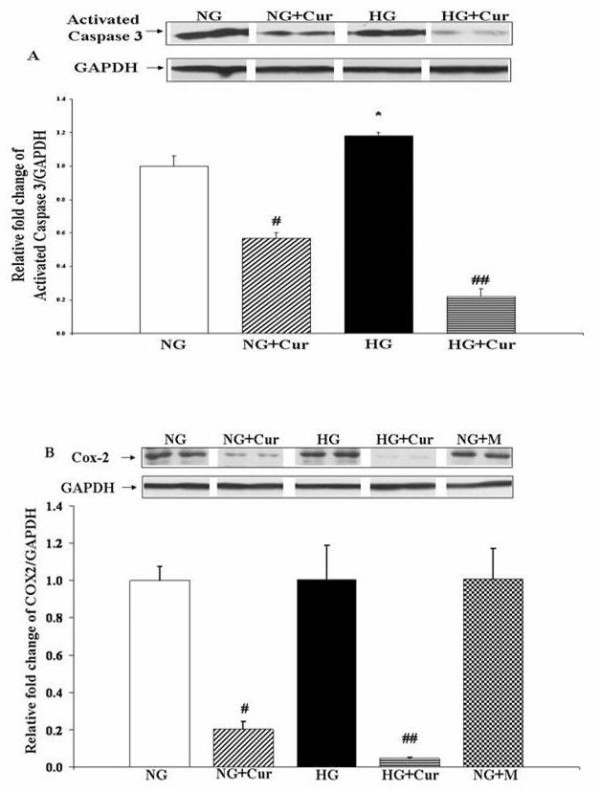
**Curcumin prevents activation of Caspase 3 and inhibits COX-2 in cultured Pods**. Representative Western blot analysis of activated Caspase 3 (a) and COX-2 (b) in Pods. 5.5 mM glucose (NG), 5.5 mM glucose+30 μM curcumin (NG+Cur), 30 mM glucose (HG), 30 mM glucose+30 μM curcumin (HG+Cur). Results were normalized with GAPDH. All data expressed as mean ± SEM (n = 3). *P < 0.05 compared with NG; #P < 0.01 compared with NG; ##P < 0.01 compared with HG.

When podocytes were harvested immediately after a 1-hr exposure to Cur_30 μM_, activation of caspase-3 was attenuated to levels significantly below those observed in the control NG conditions (1.00 ± 0.04 (NG) vs. 0.57 ± 0.02 (NG+ Cur_30 μM_); P < 0.01; Figure 2a). This inhibition was even more pronounced in HG media (1.18 ± 0.01 (HG) vs. 0.22 ± 0.03 (HG+ Cur_30 μM_); P < 0.02). Similar results were observed for the effect of Cur_30 μM _on COX-2 expression (Figure 2b) (COX-2, (1.00 ± 0.08 (NG) vs. 0.20 ± 0.05 (NG+ Cur_30 μM_); P < 0.01)). Cur had a greater effect on COX-2 inhibition in HG than NG media, (COX-2, 1.00 ± 0.18 (HG) vs. 0.05 ± 0.004 (HG+ Cur_30 μM_); P < 0.01)).

### The effect of Cur feeding on DN as measured by urine albumin/creatinine (Ualb/cr) ratio

**Figure 3:**
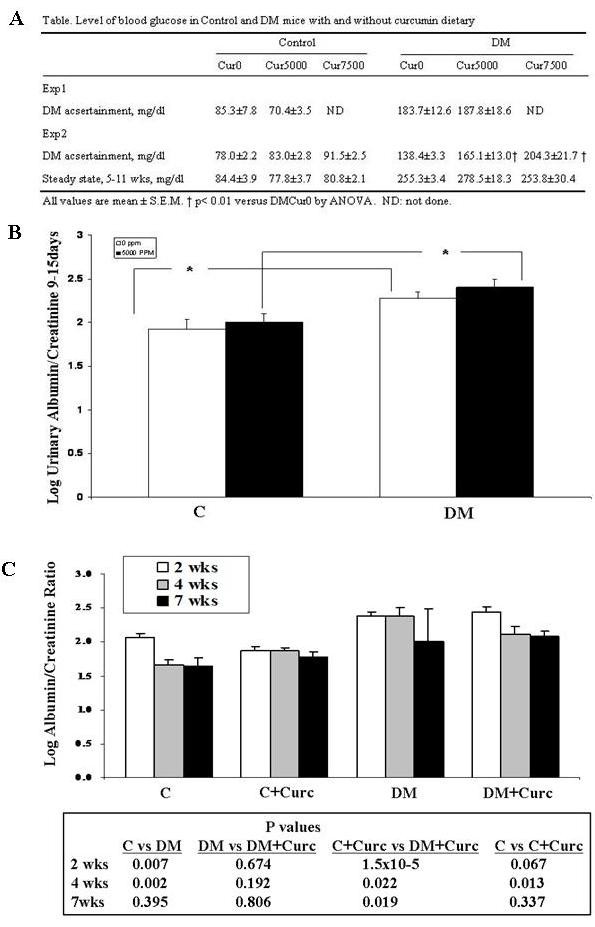
**Fasting blood glucose values and effects of curcumin on DN measured by urinary albumin/creatinine in noDM and DM mice**. (a) Fasting blood glucose in mice in Experiments 1 and 2. 24 h urine was collected to measure Ualb/cr in noDM and DM mice. (b) Ualb/cr from urine collected on days 9-15 for noDM and DM mice given control chow (0 ppm curcumin) or chow with 5,000 ppm curcumin. Mice were fed curcumin post-Stz injections (Experiment 1). (c) Ualb/cr at 2, 4 and 7 weeks for noDM and DM mice given control chow (0 ppm) or curcumin chow (data pooled for mice fed 5,000 and 7,500 ppm curcumin). Mice were fed curcumin pre-Stz injections (Experiment 2). All data are log-transformed (log_10_). *P < 0.05

Diets with Cur or identical control diets without Cur were fed to noDM mice and mice with DM beginning one week after the last Stz injection (Experiment 1). Ualb/cr was measured as an indicator of the development and severity of DN. Blood glucose in the six experimental groups one week after the last Stz injection were not different and are summarized in Figure 3a. Ualb/cr was measured after 9 and 15 days on the various diets. Since similar levels of Ualb/cr were present in the diabetic mice at 9 and 15 days, pooled data from these two time points are provided (Figure 3b). As expected, Ualb/cr excretion (log_10_) was higher in DM_Cur0 _than noDM _Cur0 _mice (2.23 ± 07 vs1.93 ± 0.11, respectively; P < 0.05), even at this early time point. (Original Ualb/cr measure was μg/mg). However, In DM_Cur5000 _mice, Cur did not lower Ualb/cr. Ualb/cr excretion in DM_Cur5000 _mice was actually higher (2.41 ± 0.09) than DM_Cur0 _mice (2.00 ± 0.09; P < 0.05).

Since the feeding regimen in Experiment 1 failed to lower Ualb/cr, we performed Experiment 2, in which Cur feeding preceded Stz DM-induction. In addition, to address the possibility that the failure to lower albuminuria in the DM mice was due to a dose effect, a Cur_7,500_diet was also studied. Thus, Experiment 2 addressed three concerns with the design of Experiment 1: 1) that the administration of Cur began too late after diabetes induction; 2) that the dose of Cur was inadequate to induce a beneficial response; and 3) that the duration of therapy was too brief to demonstrate attenuation of severity even if it did not demonstrate attenuation of induction of diabetic nephropathy. Thus, in Experiment 2, mice received either Cur_0 _diets, or identical diets with Cur_5,000 _or Cur_7,500_. Diets were begun one week prior to Stz injections to achieve a steady state of Cur prior to the induction of DM. Then, DM was induced with five daily Stz injections. DM was confirmed one week after the last Stz injection. Fasting blood glucose one week after the last of the five Stz injections in the six groups are summarized (Figure 3a), and were higher in mice fed curcumin. For this reason, additional fasting blood glucose measurements were performed in these mice and in additional mice for up to 11 weeks after Stz-diabetes induction. These values failed to confirm this trend (Figure 3a). Overnight urines for Ualb/cr were collected in weeks 2, 4, and 7 (Figure 3c). Since no difference was apparent, data from mice who received Cur_5,000 _and Cur_7,500 _were pooled. The anticipated increment in Ualb/cr excretion in DM mice compared to noDM mice was observed, both at week 2 (noDM_Cur0_, 2.07 ± 0.06 μg/mg vs. DM_Cur0_, 2.38 ± 0.07 μg/mg) and at week 4 (noDM_Cur0_, 1.65 ± 0.09 μg/mg vs. DM_Cur0_, 2.38 ± 0.12 μg/mg) (P < 0.05). However, confirming the observations in Experiment 1, even when Cur feeding began before DM induction, Cur still failed to attenuate albuminuria in the DM animals.

### Urinary curcuminoid excretion as a measure of Cur pharmacodynamics

**Figure 4:**
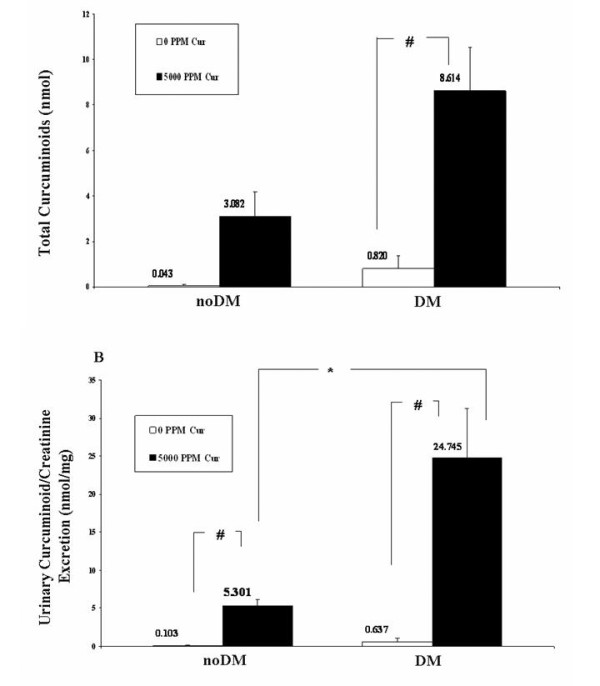
**Urinary curcuminoid excretion in noDM and DM mice fed with curcumin versus control chow**. Curcumin metabolites were measured by High Performance Liquid Chromatography (HPLC) as a measure of bioavailability in a timed urine collection. (a) Total urinary curcuminoids in nmol and (b) Urinary curcumin/creatinine in nmol/mg. All data are log-transformed (log_10_). *P < 0.05; #P < 0.01.

**Figure 5:**
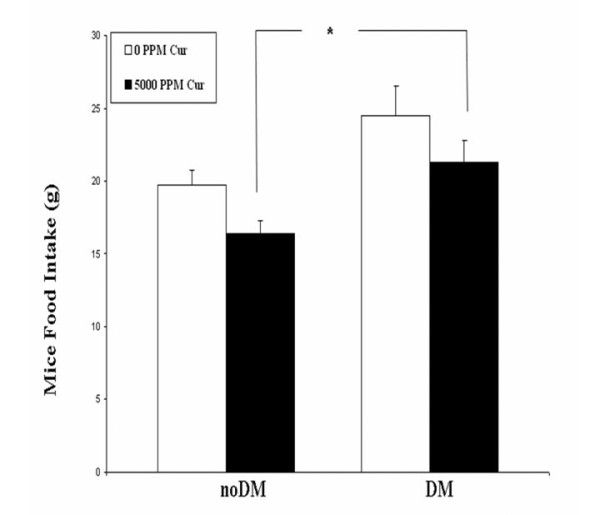
**Mouse Food Intake**. After Stz-injection and curcumin feeding began (Experiment 1), the total amount of control chow (0 ppm curcumin) or curcumin chow (5,000 ppm curcumin) consumed by each mouse over the preceding 4 days was measured on day 4. *P < 0.05.

Low bioavailability of Cur is thought to limit its potential clinical efficacy. Low plasma levels make these assays technically difficult to perform and relatively unreliable as a measure of curcumin's pharmacodynamic properties. Urinary HPLC curcuminoid measurements were therefore carried out to explore the potential use of a timed urine collection as a measure to reflect Cur pharmacodynamics. Total urine curcuminoid from a timed collection was measured in mice receiving Cur_0 _and Cur_5,000 _diets. Urine curcuminoid was expressed both as total urinary curcuminoid (Figure 4a) and also as urine cucuminoid adjusted for urine creatinine (Figure 4b). In urine samples with no Cur (eg, urine collected from mice receiving the Cur_0 _diet), an interfering substance was identified that resulted in a low level absorption value when HPLC measurements for Cur were made at 262 nm. After adjusting for this at 262 nm, there was no measurable curcuminoid in mice fed Cur_0 _diets. Urinary curcuminoid was abundantly detected in mice fed the Cur_5,000 _diet. The total urinary curcuminoid excretion in both noDM_Cur5,000 _(3.08 ± 1.09 nMol) and DM_Cur5,000 _(8.61 ± 1.93 nMol) mice was easily measurable; the levels in DM and noDM mice given the Cur_0 _chow were generally undetectable. When adjusted for urine creatinine excretion, urinary Cur/cr levels were much higher in DM_Cur5,000 _(24.74 ± 6.56 nmol/mg) compared to noDM_Cur5,000 _(5.30 ± 0.85 nmol/mg; P < 0.05) mice. This large difference can be accounted for by polyphagia and low muscle mass (and therefore low urine creatinine excretion) in the diabetic mice. DM_Cur0 _mice ingested somewhat more food than those with noDM _Cur0 _(24.5 ± 2.05 vs. 19.8 ± 1.03 gm, P = 0.10), although this difference did not reach statistical significance. DM_Cur5,000 _mice also ingested significantly more food than the noDM_Cur5,000 _group (21.3 ± 1.48 gm vs. 16.4 ± 0.93 gm, P < 0.05), but both Cur_5,000 _groups consumed less food than the Cur_0 _groups (Figure 5). Urine curcuminoid/cr excretion in DM mice was approximately four times higher than the noDM mice, but food intake was only ~50% higher. Total urine creatinine over the 12-hour collection period in the diabetic mice was 261 ± 72 μg, and in the non-diabetic control mice was 548 ± 128 μg, reflecting the lower muscle mass in the more wasted diabetic animals. Taken together, the polyphagia and the reduced muscle mass of the diabetic mice accounted for the large observed differences in the urine curcuminoid/creatinine ratio in the DM compared to noDM mice. In addition, the data show incontrovertibly that renal exposure to curcuminoid was abundant. The data demonstrate that the failure to attenuate diabetic nephropathy in the DBA2J mice was not due to a failure of the administered Cur and/or its metabolites to reach the target organ. Furthermore, these results suggest that urinary curcuminoid/cr measurements may be a reliable measure of Cur bioavailability.

### Curcumin activated renal cortical p38MAPK and reduced total HSP25 in Stz-DM mice

**Figure 6:**
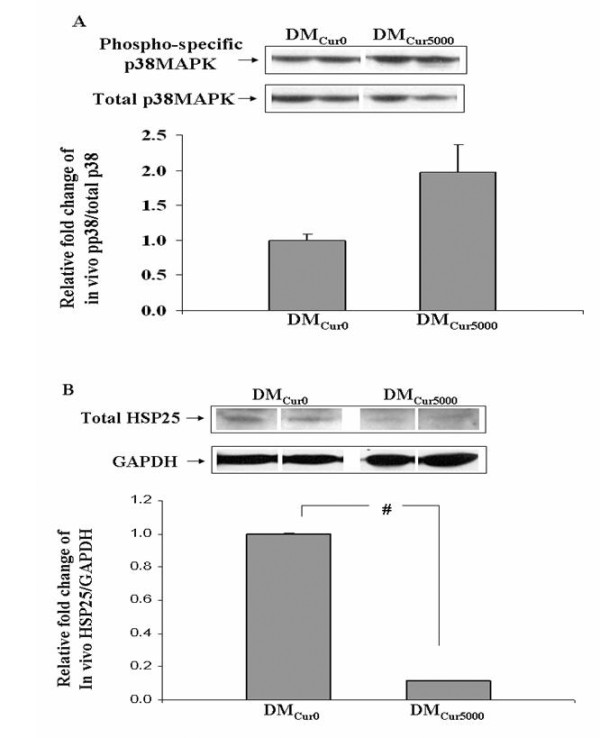
**Effects of Curcumin on p38MAPK and total HSP25 on Stz-DM mice**. Representative Western blot and quantitative evaluation of phospho-specific p38MAPK (a) and total HSP25 relative to GAPDH (b) in renal cortex of DM_Cur0 _and DM_Cur5,000 _mice. *P < 0.05; #P < 0.01.

In renal cortical samples from mice with DM for 9-15 days, curcumin feeding induced a trend toward phosphorylation of p38MAPK (DM_Cur0_, 1.0 ± 0.09 vs. DM_Cur5,000_, 2.0 ± 0.39; P = 0.07; Figure 6a) and significantly decreased total HSP25 10-fold (DM_Cur0_, 1.0 ± 0.006 vs. DM_Cur5,000_, 0.11 ± 0.004; P < 0.01; Figure 6b). These *in vivo *data, particularly those observed for HSP25, demonstrate the biological activity of curcumin in the kidney despite its failure to attenuate albuminuria.

### The effect of curcumin feeding on urinary 12-HETE/cr excretion in noDM and DM mice

**Figure 7:**
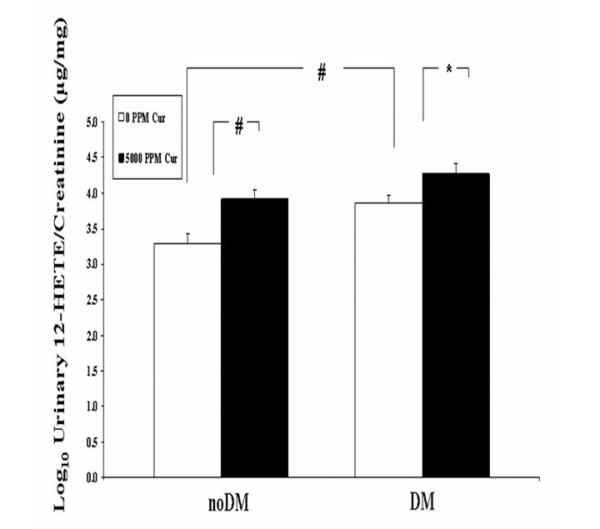
**Curcumin did not lower U 12-HETE/cr excretion in noDM and DM mice**. Urinary 12-HETE/cr ratios for noDM and DM mice fed chow with 0 and 5,000 ppm curcumin. Mice were fed curcumin post-Stz injections (Experiment 1). Graph represents pooled data (day 9 and 15). *P < 0.05; #P < 0.01.

We measured urine 12-HETE/cr in samples collected on days 9 and 15 (Figure 7). Urinary 12-HETE/cr was higher in DM than in noDM animals receiving either Cur_0 _(P < 0.01) or Cur_5,000 _chow (P = 0.14). These results are consistent with the activation of the 12/15-lipoxygenase (12/15 LO) pathway in diabetes [28]. Diabetic mice fed DM_Cur5,000 _had numerically higher urinary12-HETE/cr levels than DM_Cur0 _mice (4.27 ± 0.14 ng/mg vs 3.87 ± 0.10 ng/mg)(P < 0.05). Moreover, even in noDM mice, curcumin in the diet increased urine 12-HETE/cr (noDM_Cur5,000_, 3.92 ± 0.13 ng/mg vs noDM_Cur0 _diet, 3.30 ± 0.12 ng/mg, P < 0.01). These results further confirm the pharmacodynamic HPLC data and show that curcumin induced a renal biological effect, a conclusion also consistent with the decrement in HSP25 during curcumin feeding.

## Conclusions

Curcumin (diferuloylmethane) has anti-inflammatory, anti-oxidant, and anti-proliferative properties. It inhibits the arachidonic acid pathway, especially COX-2 [29-33]. It has been reported to maintain cytoskeletal stress fibers in cells exposed to stressors [34], and in some settings, it is cytoprotective [19,35-38]. However, in high concentrations, it is also pro-apoptotic [39]. The latter property has been exploited extensively *in vitro *and *in vivo*, and curcumin has been utilized experimentally as a potential therapy in cancer [40-46].

The *in vitro *studies reported herein are consistent with some, but not all of these observations. Our experiments show that in podocytes cultured under basal or high glucose conditions, acute exposure to curcumin induced the phosphorylation of both p38MAPK and downstream HSP25. These changes were associated with inhibition of COX-2, and a trend towards attenuation of F- to G-actin cleavage. In association with these changes, a dramatic inhibition of activated caspase-3 was observed. The pro-survival, anti-inflammatory, anti-apoptotic, and structural preservation tendencies induced by curcumin in podocytes *in vitro *could be potentially therapeutic if replicated *in vivo*. Therefore, we tested whether curcumin would diminish the albuminuria characteristic of DN in experimental animals. We measured curcumin and its metabolites in timed urine collections to verify renal curcuminoid exposure. Our findings are distinct from other publications in which benefits for DN conferred by curcumin are reported [47-50]. Curcumin administered in the diet either before or 1 week after Stz-DM in DBA2J mice failed to ameliorate albuminuria. A trend towards renal cortical p38MAPK activation was observed (p = 0.07), and total HSP25 content diminished dramatically, the latter confirming that curcumin did induce a biological effect in the kidneys. Furthermore, the anticipated decrement in 12/15-LO activation did not materialize, and urinary 12-HETE was increased in curcumin-fed diabetic mice. The decrement in total HSP25 and the increase in urine 12-HETE excretion in curcumin-fed DBA2J mice may be markers and/or mediators, at least in part, of the failure of curcumin to achieve an anti-albuminuric effect in these diabetic mice.

In our *in vitro *studies, the most surprising result was the attenuation of the apoptosis marker, activated caspase-3, along with an increase in phosphorylation of p38MAPK in curcumin-treated podocytes. Activation of p38MAPK has been shown to induce apoptosis predominantly in cultured neoplastic cells [44,45,51-58], but also in non-neoplastic cells [59-63]. However, p38MAPK activation is not always pro-apoptotic in experimental settings, and it is cytoprotective in some cells and circumstances. For instance, in human colonic carcinoma cells, inhibition of p38MAPK activity with SB203580 increased drug-induced apoptosis [64]. In addition, in a model of anoxia-reoxygenation-induced lung endothelial cell apoptosis, SB203580 or transfection with a p38α dominant negative mutant each inhibited the anti-apoptotic effects of carbon monoxide through a pathway involving the modulation of caspase 3 [65]. Thus, the relationship between p38MAPK activation and apoptosis may be dependent on cell type and the inciting stimulus, and both apoptosis and cytoprotection have been observed [38,57,64,65]. Our *in vitro *data suggest that in curcumin-stimulated podocytes under the conditions of study, the p38MAPK-HSP25-apoptosis axis favored cytoprotection, consistent with a minority of published reports in the literature. Since phosphorylated p38MAPK is one of the major regulators of the phosphorylation of downstream HSP25, activation of the p38MAPK-HSP25 pathway may explain both the tendency towards maintenance of the actin cytoskeleton and the attenuation of apoptosis in this *in vitro *model.

HSPs are a pleiotropic family of chaperone proteins with numerous functions. Phosphorylated HSP25 monomers play a role in the maintenance of the actin cytoskeleton during cell stress [11,13,66-69]. One group recently reported that the anti-apoptotic properties of HSP25/27 were ascribable to its maintenance of actin cytoskeletal integrity, which prevented mitochondria from releasing cytochrome c [70]. Actin cytoskeletal disruption may be both a marker and a mechanism of apoptosis [71-73]. Through the stabilization of actin, phosphorylated HSP25 may attenuate apoptosis. The anti-apoptotic activities of phosphorylated HSP25/27 in cells exposed to TNFα have also been ascribed to enhanced IKKγ proteasomal degradation, which increases NFκB activity in some cells [74-76], but not in others [77]. Non-phosphorylated HSP25 oligomers also enhance cell survival, but through mechanisms involving the inhibition of canonical targets in the mitochondrial and death-domain apoptotic pathways and through inhibition of NFκB activation [74-81]. While curcumin increases HSP70 [19-22], limited information is available concerning the effect of curcumin on HSP25/27. Curcumin increased total HSP27 in glioma cells cultured under stress-conditions by prolonging the stress-induced activation of the heat shock element-binding activity of heat shock transcription factor [82]. In *in vivo *studies, these same investigators also showed further induction of HSP25 by curcumin in the adrenal glands and livers of rats exposed to heat stress. In contrast, in our *in vitro *studies in curcumin-treated podocytes, phosphorylated HSP25 was increased, but not total HSP25 (not shown). Since phosphorylated HSP25 regulates the maintenance of the actin cytoskeleton and NFκB activation, our *in vitro *data are consistent with a role for activation of the p38MAPK-HSP25 pathway in the observed trend favoring maintenance of stress fibers in curcumin-treated podocytes during high glucose exposure. In other published experiments consistent with these findings, curcumin has been reported to increase stress fibers and F-actin in prostate cancer cells [34]. Thus, the increase in phosphorylated HSP25 induced by curcumin *in vitro *may contribute to the observed curcumin-associated trend to maintain actin stress fibers and the decrement in activated caspase-3.

Finally, curcumin inhibited COX-2 *in vitro*. Curcumin is well-known to inhibit the arachidonic acid pathway, particularly COX-2 [30-33,83-86]. Our *in vitro *results showing inhibition of COX-2 by curcumin is consistent with these other published studies. Medicinal COX-2 inhibitors such as celecoxib induce apoptosis, but COX-2 inhibition by other means, including molecular interventions, do not necessarily induce apoptosis [87]. Taken together, our *in vitro *data demonstrate that in podocytes cultured in normal or high glucose media, curcumin activates the p38MAPK-HSP25 pathway, inhibits COX-2, attenuates apoptosis, and likely contributes towards the trend for cytoskeletal maintenance.

In contrast to our findings *in vitro*, which corroborate other published findings, our inability to demonstrate a benefit for curcumin in diabetic nephropathy (DN) in DBA2J mice is unique among published studies in this field. We were unable to show an anti-albuminuric effect or an attenuation in urine 12-HETE excretion in diabetic DBA2J mice, despite our clear ability to demonstrate renal exposure to curcuminoids by measuring curcumin and its metabolites in urine. Curcumin has previously been reported to inhibit proteinuria, albuminuria, and/or histologic change in Stz-DN in rats [48-50]. Species, strain, and/or dosing differences may underlie our inability to demonstrate a clinical benefit from curcumin in mice while others reported benefit in rats. Indeed, in a recently published paper, Li et al [88] showed that in the DBA2J mouse used herein, which has a naturally occurring mutation in the gene glycoprotein non-metastatic melanoma protein b (gpnmb), there is a defect in renal reparative processes. It is possible that the negative results observed for curcumin in this mouse are due to this inherited reparative defect. It is well-known that both susceptibility to disease and responsiveness to therapy are influenced by genetic predisposition." However, review of the publications in which benefit from curcumin was actually reported raises some skepticism concerning the robustness of these observations. In the work by Babu et al in Stz-DN in Wistar rats, renal hypertrophy, measures of tubular proteinuria, urine excretion of proteins with MW > 66 kD, and histological change were improved at 8 weeks [49]. Of note, the investigators went to great lengths to publish the results of a large number of tubular and large molecular weight proteinuria markers, but did not publish their albuminuria result. In addition, blood glucose data are not provided, a description of how the histologic analyses were performed is lacking, and the photomicrographs provided are of very low magnification and not easily interpretable by the reader. In the work by Sharma et al [48], a claim for the benefit of curcumin on DN was based on lower albuminuria concentration (but not lower urine albumin/creatinine ratio or albumin/unit time, which are the standard methods of reporting); lower serum creatinine and urea nitrogen; higher creatinine clearance; and less renal pathology in the curcumin-treated diabetic rats compared to diabetic rats on a control diet. Unfortunately, in this experiment, the curcumin-treated rats had lower plasma glucose levels than the diabetic rats receiving a control diet. The authors attribute this to the curcumin treatment itself. Nevertheless, the difference in glycemic control confounds the interpretation of the role of curcumin in directly ameliorating DN in this experiment. Furthermore, the time point of study (6 weeks after diabetes induction) was shorter than optimal for the establishment of DN in rats, and the histologic sections provided are of inadequate quality, falling short of establishing DN changes. In the work by Chiu et al [47], Sprague-Dawley rats with Stz-DN were studied after 4 weeks. Curcumin-treated rats had improvement in a number of biochemical parameters including attenuation of renal mRNAs for fibronectin, eNOS, TGF-beta, heme oxygenase-1, and improvements in glomerular nitrotyrosine, 8-OHdG, transcription coactivator p300, and NFκB. Albuminuria was not measured, the studies were not carried out beyond 4 weeks, and, as pointed out by others [89], key controls for the ethanol and DMSO diluents were lacking. In the work by Tikoo et al [50], Stz-DN was studied in Sprague-Dawley rats after 8 weeks, having received curcumin treatment for 6 weeks. Improvement in DN was inferred from modulation in the curcumin-treated group of blood urea nitrogen, serum creatinine, and kidney/body weight ratio. Acceptance of this conclusion is limited by concerns regarding the use of BUN and serum creatinine in polyuric animals with low muscle mass as robust measures of renal function, the methodology used for the measure of serum creatinine (picric acid, [90]), and the absence of a measure of albuminuria. Taken together, no prior report showing a beneficial effect of curcumin on Stz-DN actually measured the urine albumin/creatinine ratio, a cardinal manifestation of DN, and many of the studies had other significant design flaws. The work reported herein is the only one to date to demonstrate pharmacodynamic data consistent with renal exposure to curcumin and its metabolites, biochemical changes consistent with a renal biological effect of curcumin, but no ameliorative effect on albuminuria, which is the key clinical feature of early DN.

The burden of explaining why curcumin failed to ameliorate albuminuria in these mice remains, and one can only speculate. A unique response in this mouse strain cannot be ruled out, as it is well-appreciated that genetic backgrounds influence both disease susceptibility and response to treatments. In addition, at least in Experiment 2, fasting blood glucose was higher at week 1 in mice receiving curcumin, a finding that was not replicated in measures taken at later weeks. These early differences were statistically significant, but their biological significance is uncertain. Nevertheless, we cannot exclude that this apparently transient and relatively small increment in blood glucose early in disease development contributed to the lack of apparent efficacy of curcumin to attenuate albuminuria. However, some biological observations may be relevant. We have previously shown in podocytes cultured under normal or high glucose conditions, and in renal cortical tissue from diabetic and control rats, that phosphorylated HSP25 appears as an acute adaptation to glycemic stress. This adaptation was associated with maintenance of the podocyte cytoskeleton *in vitro*, and an association with normoalbuminuria *in vivo*. Decrements in phospho-HSP25 later in the course of Stz-DN were associated with the appearance of albuminuria and glomerular podocyte effacement [18]. We have also reported that in mice overexpressing HSP27, there was diminished beta cell apoptotic death from Stz and an attenuation of Stz-DN. In other studies *in vitro*, direct binding of HSP25/27 to the upstream regulator of NFκB, IKKγ (NEMO), inhibited pancreatic beta cell apoptosis [74-77]. These data underscore the significant relationship between phosphorylated and non-phosphorylated HSP25/27 with cytoskeletal maintenance and cytoprotection from apoptotic death. Taken together, the experimental results published herein are consistent with the hypothesis that the profound decrease in total HSP25 in the curcumin-treated Stz-DN mice may confer a susceptibility to loss of structural cellular integrity and apoptosis of cells comprising the glomerular capillary wall, resulting in albuminuria. In Tikoo et al, a decrement in renal HSP25 is also reported in Stz-DN rats fed with curcumin [50].

Finally, the failure to mitigate albuminuria in the curcumin-treated Stz-DN mice may have been related to the persistent activity of the arachidonic acid pathway enzyme, 12/15-LO. Natarajan et al showed that the 12/15-LO pathway mediates the actions of the key effector molecules that induce albuminuria and extracellular matrix accumulation in DN, including glucose, TGF-beta, angiotensin II, and PDGF in vascular smooth muscle cells [91]. Our prior work showed that 12/15-LO mRNA and protein are induced in mesangial cells in HG and in Stz-DN rat glomeruli [6,92-94]. In podocytes *in vitro *and in Stz-DN, HG stimulated 12/15-LO mRNA and protein synthesis, podocyte p38MAPK activation, and collagenα5(IV) mRNA and protein, while 12/15-LO inhibition diminished HG-stimulated podocyte collagenα5(IV) mRNA and protein [6].

Curcumin has been reported to inhibit lipoxygenases by one group of investigators [95], but was found to be a substrate of lipoxygenases by another group [96]. Urine 12-HETE is a reliable measure of activation of the 12/15-LO pathway *in vivo *[97], and in these curcumin-treated mice, the urine 12-HETE/cr ratio was increased. In prior studies performed to inhibit 12/15-LO in Stz-DN rats, our published work showed that chemical inhibition of 12/15-LO is only transiently effective, and that tachyphylaxis occurs rapdily [98]. In the rats receiving the chemical inhibitor, a linear relationship between urine 12-HETE excretion and albuminuria was observed [97]. The failure of curcumin to suppress activation of 12/15-LO, as evidenced by the high urine 12-HETE/cr ratio, may have contributed to the albuminuria observed in the curcumin-treated diabetic DBA2J mice.

Thus, our data extend and confirm prior *in vitro *evidence concerning the effects of curcumin on cultured cells exposed to glycemic stress. In cultured podocytes, curcumin induced the phosphorylation of p38MAPK and downstream HSP25, inhibited COX-2 and the activation of caspase-3, and demonstrated a tendency to attenuate F-actin cleavage to G-actin monomers. However, in DBA2J mice with Stz-DM, despite pharmacodynamic proof of exposure to orally administered curcumin by timed urine collections measuring excreted curcuminoids, curcumin attenuated the HSP25 response to Stz-DM, increased urinary 12-HETE excretion, and failed to attenuate the albuminuria of DN. While strain, species, and dosing issues may be responsible for this negative result, the biological responses of HSP25 and 12/15-LO to curcumin may underlie this failure. Thus, despite encouraging *in vitro *effects, these data do not confirm prior published *in vivo *work and suggest that curcumin is not universally useful in ameliorating DN. In addition, these studies suggest that timed urine collections may be useful for monitoring curcumin dosing and renal pharmacodynamics.

## Competing interests

The authors declare that they have no competing interests.

## Authors' contributions

JM and LP carried out the molecular studies, animal experiments, data analysis and drafted the manuscript. YW carried out molecular studies and performed data analysis in the revision manuscript. TD and RN participated in the design of the study. JL participated in animal experiments. SA conceived of the study, and participated in its design and coordination and wrote the manuscript. All authors read and approved the final manuscript.

## Pre-publication history

The pre-publication history for this paper can be accessed here:

http://www.biomedcentral.com/1472-6882/10/67/prepub
